# Phenotype prediction for mucopolysaccharidosis type I by in silico analysis

**DOI:** 10.1186/s13023-017-0678-1

**Published:** 2017-07-04

**Authors:** Li Ou, Michael J. Przybilla, Chester B. Whitley

**Affiliations:** 1 Gene Therapy Center, Department of Pediatrics, University of Minnesota, Minneapolis, MN 55455 USA; 2 Department of Genetics, Cell Biology and Development, University of Minnesota, Minneapolis, MN 55455 USA

**Keywords:** In silico, Single nucleotide polymorphism, Genotype/phenotype correlation, Mucopolysaccharidosis

## Abstract

**Background:**

Mucopolysaccharidosis type I (MPS I) is an autosomal recessive disease due to deficiency of α-L-iduronidase (IDUA), a lysosomal enzyme that degrades glycosaminoglycans (GAG) heparan and dermatan sulfate. To achieve optimal clinical outcomes, early and proper treatment is essential, which requires early diagnosis and phenotype severity prediction.

**Results:**

To establish a genotype/phenotype correlation of MPS I disease, a combination of bioinformatics tools including SIFT, PolyPhen, I-Mutant, PROVEAN, PANTHER, SNPs&GO and PHD-SNP are utilized. Through analyzing single nucleotide polymorphisms (SNPs) by these in silico approaches, 28 out of 285 missense SNPs were predicted to be damaging. By integrating outcomes from these in silico approaches, a prediction algorithm (sensitivity 94%, specificity 80%) was thereby developed. Three dimensional structural analysis of 5 candidate SNPs (P533R, P496R, L346R, D349G, T374P) were performed by SWISS PDB viewer, which revealed specific structural changes responsible for the functional impacts of these SNPs. Additionally, SNPs in the untranslated region were analyzed by UTRscan and PolymiRTS. Moreover, by investigating known pathogenic mutations and relevant patient phenotypes in previous publications, phenotype severity (severe, intermediate or mild) of each mutation was deduced.

**Conclusions:**

Collectively, these results identified potential candidate SNPs with functional significance for studying MPS I disease. This study also demonstrates the effectiveness, reliability and simplicity of these in silico approaches in addressing complexity of underlying genetic basis of MPS I disease. Further, a step-by-step guideline for phenotype prediction of MPS I disease is established, which can be broadly applied in other lysosomal diseases or genetic disorders.

**Electronic supplementary material:**

The online version of this article (doi:10.1186/s13023-017-0678-1) contains supplementary material, which is available to authorized users.

## Background

Mucopolysaccharidosis type I (MPS I) is a lysosomal disease included within the genetically heterogeneous group of mucopolysaccharidoses (MPSs). MPS I results from mutations in the gene encoding the lysosomal enzyme α-L-iduronidase (IDUA; glycosaminoglycan a-L-iduronohydrolase, OMIM 252800) [1]. Deficiency of IDUA leads to progressive lysosomal accumulation of glycosaminoglycans (GAG) heparan and dermatan sulfate in tissues. Based on the severity of symptoms, MPS I can be divided into three subtypes, from mild (Scheie syndrome, OMIM 607016) to intermediate (Hurler-Scheie syndrome, OMIM 607015) to severe (Hurler syndrome, OMIM 607016). Scheie or Hurler-Scheie patients have symptoms including growth delay, aortic valvular disease, skeletal dysplasias, corneal clouding and joint stiffness. In addition to having these symptoms, but in a more pronounced way, Hurler patients also have growth delay, hepatosplenomegaly, coarse facial features, hydrocephalus, mental retardation and neurodegeneration.

It has been shown that the earlier enzyme replacement therapy or hematopoetic stem cell transplantation is performed, the better the outcome is [2–5]. Since early initiation of treatment is more likely to improve clinical outcomes, early diagnosis and accurate phenotype prediction are essential. However, genotype/phenotype correlation of MPS I has not been well established [6, 7]. To date, assessment of the phenotype is generally based on clinical signs and symptoms. A recent study showed a lack of consensus on the assessment of phenotypic severity solely based on signs and symptoms at presentation [8]. Therefore, establishment of a reliable and easy-to-use phenotype prediction method based on genotype will be of great benefit.

The single nucleotide polymorphisms (SNPs) are the most common form of genetic mutations. SNP was originally defined as a single nucleotide variant with a frequency in genome of more than 1% [9]. In this study, for the simplicity of description, single nucleotide variants with a frequency of less than 1% were also included in the analysis. While many SNPs are phenotypically neutral, others could cause disease, predispose human to disease, or influence response to medicine. Previous studies on polymorphisms screening by in silico analysis contributed to predicting the functional non-synonymous SNPs (nsSNPs) in genes such as G6PD [10], ATM [11], PTEN [12], BRAF [13] and BUB1B [14]. This powerful computed methodology enables prioritizing SNPs with functional significance from a large quantity of neutral non-risk variants. To date, computational analyses of IDUA gene for phenotype prediction have not been performed. To this end, a number of bioinformatics tools, based on recent findings from evolutionary biology, protein structure research, machine learning and computational biology, may provide useful information for assessing the functional impacts of SNPs. A stepwise guideline for phenotype prediction based on genotype is established, which will benefit early diagnosis and proper treatment allocation for MPS I patients.

## Methods

### Dataset

The SNPs information (Protein accession number and SNP ID) of the IDUA gene was retrieved from the NCBI dbSNP (http://www.ncbi.nlm.nih.gov/snp/). Known disease-associated mutations in IDUA gene were retrieved from The Human Gene Mutation Database (http://www.hgmd.cf.ac.uk/ac/index.php).

### SIFT

SIFT (Sorting Intolerant From Tolerant; http://sift.jcvi.org/) can predict the effect of amino acid substitution on protein function, and classify it as ‘tolerated’ or ‘deleterious’ [15]. SIFT applies multiple alignment information for the query sequence and predicts whether substitutions are ‘tolerated’ or ‘deleterious’ by calculating the tolerance index score (0 to 1). Tolerance index score is a normalized probability that an amino acid substitution is tolerated. Substitutions with a tolerance index less than 0.05 are predicted to be ‘deleterious’ and those with greater than or equal to 0.05 are predicted as ‘tolerated’. The analysis was performed using the default settings.

### PolyPhen

PolyPhen (Polymorphism Phenotyping; http://genetics.bwh. harvard.edu/pph2/) is a probabilistic classifier which predicts the functional impacts of SNPs. PolyPhen calculates position-specific independent count (PSIC) scores for every substitution and estimates the difference between the variant scores. Based on PSIC, Polyphen classifies SNPs into ‘probably damaging’ (score > 0.85), ‘possibly damaging’ (score > 0.15) and ‘benign’ (the rest) [16].

### I-Mutant

I-Mutant (http://folding.biofold.org/cgi-bin/i-mutant2.0) is a neural-network-based web server for the automatic prediction of protein stability changes upon single amino acid substitution. I-Mutant performs analyses based on the protein sequence combined with mutational position. The output is the predicted free energy change (DDG), which classifies the prediction into: ‘large decrease’ (DDG < −0.5 kcal/mol), ‘large increase’ (DDG > 0.5 kcal/mol), or ‘neutral’ (−0.5 < DDG < 0.5 kcal/mol) [17].

### PROVEAN

PROVEAN (Protein Variation Effect Analyzer; http://provean.jcvi.org) is a sequence based predictor that estimates the impact of protein sequence variation on protein function [18]. In PROVEAN, BLAST hits with more than 75% global sequence identity are clustered together, and top 30 such clusters from a supporting sequence are averaged within and across clusters to generate the final score. A protein variant is predicted to be ‘deleterious’ if the final score is below −2.5, and is predicted to be ‘neutral’ otherwise.

### PANTHER

PANTHER (http://www.pantherdb.org/) is a database which contains a collection of protein families and subfamilies that predict the occurrence of an amino acid at a position in a family of evolutionarily related protein [19]. PANTHER uses hidden Markov model (HMM) based statistical modeling methods and multiple sequence alignments to perform evolutionary analysis of coding nsSNPs. By calculating the substitution position-specific evolutionary conservation score (subPSEC) based on an alignment of evolutionarily related proteins, PANTHER estimates the likelihood of a particular nsSNP causing a functional impact. Based on subPSEC scores, PANTHER classifies SNPs as ‘deleterious’ (score < −3) or ‘neutral’ (score > −3).

### SNPs&GO

SNPs&GO (Single Nucleotide Polymorphism Database & Gene Ontology; http://snps.biofold.org/snps-and-go/snps-and-go.html) is an support vector machine (SVM) based method used to predict the disease related mutations from protein sequences with a scoring accuracy of 82% and Matthews correlation coefficient of 0.63. For SNPs&GO, FASTA sequence of whole protein is considered to be an input option and output will be the prediction results based on the discrimination among ‘disease’ and ‘neutral’ variations of protein sequence. The probability score higher than 0.5 is defined as ‘disease’ [20].

### PHD-SNP

PHD-SNP (Predictor of Human Deleterious Single Nucleotide Polymorphisms; http://snps.biofold.org/phd-snp/phd-snp.html) is an SVM-based classifier, trained over a million amino acid polymorphism datasets using supervised training. PHD-SNP predicts whether the given amino acid substitution leads to ‘disease’ or ‘neutral’ along with the reliability index score [21].

### NetSurfP

NetSurfP (http://www.cbs.dtu.dk/services/NetSurfP/) is a web server that predicts the surface accessibility and secondary structure of amino acids. The reliability of this NetsurfP is given in the form of Z-score. The Z-score highlights the surface prediction reliability, but not associated with secondary structure [22].

### Modeling of mutant protein structures

The Swiss-PDB Viewer, a free molecular graphics program was used for viewing the modeled structures and for calculation of the root mean square deviation (RMSD) between the native and mutant structures. Swiss-PDB viewer named as Deep View, a stand-alone program, was used as an analytical tool for macromolecules [23]. To superimpose protein structures, the “Magic Interactive Fit” command was used for detection of a stretch of similar residues at sequence level to obtain a structural fit between the two models. Energy minimization for three-dimensional (3D) structures was performed using NOMAD-Ref server (http://lorentz.immstr.pasteur.fr/nomad-ref.php) [24]. Conjugate gradient method was used for energy minimization of the 3D structures.

### Project HOPE

Project Have yOur Protein Explained (HOPE; http://www.cmbi.ru.nl/hope/home) is an easy-to-use web service that analyzes the structural effects of a point mutation in a protein sequence. HOPE provides the 3D structural visualization of mutated proteins by using UniProt and DAS prediction servers. HOPE server predicts the output in the form of structural variation between mutant and wild type residues [25].

### UTRscan

UTRscan (http://itbtools.ba.itb.cnr.it/utrscan) is a web server that can analyze the untranslated regions (5′ UTR and 3′ UTR) of eukaryotic mRNA which are involved in many post-transcriptional regulatory pathways that control mRNA localization, stability and translation [26]. The internet resource for UTR analysis are UTRdb, which contains experimentally proven biological activity of functional pattern of UTR sequence from eukaryotic mRNAs. If different sequences for each UTR SNP are found to have different functional patterns, that particular UTR SNP is predicted to have functional significance.

### PolymiRTS

PolymiRTS database (http://compbio.uthsc.edu/miRSNP/) was used specifically for the analysis of SNPs in the 3′ UTR. The polymorphic microRNA target sites are classified into four classes [27]. Specifically, class ‘D’ may cause loss of normal repression, and class ‘C’ may cause abnormal gene repression control. Therefore, these two classes of PolymiRTS are most likely to have functional impacts.

## Results

### Analysis of missense SNPs using a combination of bioinformatics tools

Polymorphisms in the IDUA gene were retrieved from NCBI dbSNP database. Non-synonymous SNPs (nsSNPs) from the coding region, and untranslated (5’and 3′) region were selected for further analysis. The impacts of any amino acid substitution with its functional significance and physical properties can be determined using SIFT by aligning homologous and orthologous protein sequence. A total of 285 missense SNPs of IDUA gene were analyzed using SIFT. Out of 285 SNPs, 201(71%) were predicted to be ‘deleterious’ (tolerance index <0.05), while 157 (55%) were ‘highly deleterious’ (tolerance index = 0). All 201 SNPs predicted to be ‘deleterious’ by SIFT were further analyzed by PolyPhen. For every input SNP, Polyphen calculates PSIC score and perform BLAST query to identify homologous protein. A total of 149 SNPs were predicted to be ‘probably damaging’. For further confirmation, the PolyPhen results were subjected to I-Mutant, which is a routine SNP prediction tool based on neural network, for adding another layer of confirmation. I-Mutant estimates the effect of substitution on protein stability by calculating the reliability index (25 °C, pH 7.0). Out of 149 missense SNPs analyzed, 107 (72%) were predicted to cause ‘large decrease’, while 42 were predicted to cause ‘neutral stability’. The remaining 107 SNPs were analyzed by PROVEAN, yielding 93 deleterious and 14 neutral SNPs. Therefore, 93 out of 285 SNPs were predicted to be damaging by 4 different methods and summarized in Table 1.

**Table 1:** List of 91 nsSNP predicted as damaging by SIFT, PolyPhen, I-Mutant, PROVEAN

SNP ID	AA change	SIFT	Score	PolyPhen	Score	I-Mutant	Score	PROVEAN	Score
rs121965021	P533R	Deleterious	0	Probably damaging	1	Large decrease	−0.75	Deleterious	−7.1
rs121965029	R89Q	Deleterious	0	Probably damaging	1	Large decrease	−0.73	Deleterious	−3.08
rs121965030	A300T	Deleterious	0	Probably damaging	0.999	Large decrease	−0.77	Deleterious	−3.68
rs121965031	R619G	Deleterious	0	Probably damaging	0.999	Large decrease	−1.51	Deleterious	−4.63
rs121965033	L346R	Deleterious	0	Probably damaging	1	Large decrease	−1.77	Deleterious	−5.3
rs138731804	A160T	Deleterious	0	Probably damaging	1	Large decrease	−0.67	Deleterious	−3.31
rs140294059	C205S	Deleterious	0.04	Probably damaging	0.964	Large decrease	−1.09	Deleterious	−7.57
rs147353014	L237H	Deleterious	0	Probably damaging	1	Large decrease	−1.98	Deleterious	−6.4
rs148789453	L238Q	Deleterious	0	Probably damaging	1	Large decrease	−2.05	Deleterious	−5.33
rs183347428	D223N	Deleterious	0	Probably damaging	1	Large decrease	−1.01	Deleterious	−3.03
rs200448421	R628P	Deleterious	0	Probably damaging	0.999	Large decrease	−0.72	Deleterious	−3.86
rs201268637	R263W	Deleterious	0	Probably damaging	0.995	Large decrease	−0.71	Deleterious	−5.39
rs202051939	S269C	Deleterious	0	Probably damaging	1	Large decrease	−0.62	Deleterious	−3.94
rs368241547	F247 L	Deleterious	0.02	Probably damaging	0.993	Large decrease	−1.28	Deleterious	−4.33
rs368454909	D349N	Deleterious	0	Probably damaging	1	Large decrease	−0.78	Deleterious	−4.64
rs369090960	G265R	Deleterious	0	Probably damaging	1	Large decrease	−0.66	Deleterious	−7.46
rs371397270	D349G	Deleterious	0	Probably damaging	1	Large decrease	−1.08	Deleterious	−6.43
rs373037758	L256P	Deleterious	0	Probably damaging	1	Large decrease	−1.98	Deleterious	−6.1
rs373342547	F143 L	Deleterious	0.05	Probably damaging	1	Large decrease	−0.69	Deleterious	−4.27
rs374699130	A319T	Deleterious	0	Probably damaging	1	Large decrease	−0.52	Deleterious	−3.78
rs374779600	P533A	Deleterious	0	Probably damaging	1	Large decrease	−1.03	Deleterious	−6.51
rs374779600	P533S	Deleterious	0	Probably damaging	1	Large decrease	−1.18	Deleterious	−6.51
rs375300630	G244D	Deleterious	0	Probably damaging	1	Large decrease	−0.89	Deleterious	−5.7
rs376573681	I272T	Deleterious	0	Probably damaging	1	Large decrease	−2.04	Deleterious	−4.43
rs398123253	W434C	Deleterious	0	Probably damaging	1	Large decrease	−1.19	Deleterious	−7.94
rs527336882	L365 V	Deleterious	0	Probably damaging	1	Large decrease	−1.59	Deleterious	−2.65
rs537047205	D119A	Deleterious	0	Probably damaging	0.993	Large decrease	−0.6	Deleterious	−4.43
rs546808806	P377L	Deleterious	0	Probably damaging	0.996	Large decrease	−0.62	Deleterious	−7.86
rs546933529	G253C	Deleterious	0	Probably damaging	1	Large decrease	−1.21	Deleterious	−6.19
rs555091763	I283T	Deleterious	0	Probably damaging	0.996	Large decrease	−1.76	Deleterious	−4.22
rs558683362	M133I	Deleterious	0	Probably damaging	0.997	Large decrease	−0.61	Deleterious	−3.49
rs564306004	G84S	Deleterious	0	Probably damaging	1	Large decrease	−1.25	Deleterious	−4.42
rs587779401	Y625C	Deleterious	0	Probably damaging	1	Large decrease	−1.41	Deleterious	−5.42
rs74385837	L237F	Deleterious	0	Probably damaging	1	Large decrease	−1.12	Deleterious	−3.6
rs746018077	F495 L	Deleterious	0	Probably damaging	0.977	Large decrease	−0.82	Deleterious	−4.36
rs746606129	Q328H	Deleterious	0	Probably damaging	1	Large decrease	−2.16	Deleterious	−4.27
rs746766617	N348 K	Deleterious	0	Probably damaging	0.994	Large decrease	−0.64	Deleterious	−4.96
rs747827435	N350D	Deleterious	0	Probably damaging	1	Large decrease	−0.6	Deleterious	−4.55
rs748239393	F287C	Deleterious	0	Probably damaging	1	Large decrease	−1.52	Deleterious	−6.72
rs748589618	L216P	Deleterious	0	Probably damaging	1	Large decrease	−1.61	Deleterious	−6.03
rs749645656	D477G	Deleterious	0	Probably damaging	0.999	Large decrease	−1.54	Deleterious	−4.93
rs750230093	R255W	Deleterious	0	Probably damaging	1	Large decrease	−0.56	Deleterious	−6.87
rs750496798	R363C	Deleterious	0	Probably damaging	1	Large decrease	−0.82	Deleterious	−7.39
rs750893089	P309T	Deleterious	0	Probably damaging	1	Large decrease	−1.26	Deleterious	−5.62
rs751396984	R383G	Deleterious	0	Probably damaging	1	Large decrease	−1.15	Deleterious	−5.08
rs751547595	A367T	Deleterious	0	Probably damaging	0.976	Large decrease	−0.92	Deleterious	−3.39
rs751676744	V88F	Deleterious	0	Probably damaging	1	Large decrease	−1.29	Deleterious	−3.65
rs751792135	G78D	Deleterious	0	Probably damaging	0.998	Large decrease	−0.96	Deleterious	−3.77
rs751861062	A204T	Deleterious	0	Probably damaging	1	Large decrease	−0.86	Deleterious	−3.67
rs752529809	P385S	Deleterious	0	Probably damaging	0.997	Large decrease	−1.37	Deleterious	−6.99
rs753308650	G168R	Deleterious	0	Probably damaging	1	Large decrease	−0.69	Deleterious	−7.18
rs753875643	P232T	Deleterious	0	Probably damaging	1	Large decrease	−1.31	Deleterious	−6.77
rs753905054	D570G	Deleterious	0	Probably damaging	1	Large decrease	−0.81	Deleterious	−4.43
rs754154200	E182K	Deleterious	0	Probably damaging	1	Large decrease	−0.88	Deleterious	−3.76
rs754674352	P128S	Deleterious	0	Probably damaging	1	Large decrease	−1.39	Deleterious	−6.2
rs754681846	R368C	Deleterious	0	Probably damaging	0.976	Large decrease	−0.81	Deleterious	−6.8
rs754876066	T194P	Deleterious	0	Probably damaging	0.999	Large decrease	−0.96	Deleterious	−4.7
rs754949360	R383H	Deleterious	0	Probably damaging	1	Large decrease	−1.12	Deleterious	−3.8
rs757171895	G208S	Deleterious	0	Probably damaging	1	Large decrease	−1.19	Deleterious	−5.77
rs757706461	P183S	Deleterious	0	Probably damaging	1	Large decrease	−1.44	Deleterious	−7.18
rs758452450	A75T	Deleterious	0	Probably damaging	1	Large decrease	−0.63	Deleterious	−2.58
rs760900176	P229L	Deleterious	0	Probably damaging	0.963	Large decrease	−0.63	Deleterious	−5.85
rs762037549	E582K	Deleterious	0	Probably damaging	1	Large decrease	−0.66	Deleterious	−2.74
rs762623046	R166T	Deleterious	0	Probably damaging	0.975	Large decrease	−0.96	Deleterious	−4.06
rs764882035	V254G	Deleterious	0	Probably damaging	0.999	Large decrease	−3.19	Deleterious	−4.6
rs766030255	T179S	Deleterious	0	Probably damaging	0.957	Large decrease	−0.58	Deleterious	−2.96
rs766033352	I259M	Deleterious	0	Probably damaging	1	Large decrease	−1.73	Deleterious	−2.7
rs76722191	V322E	Deleterious	0	Probably damaging	1	Large decrease	−0.81	Deleterious	−5.5
rs768389832	P54S	Deleterious	0	Probably damaging	1	Large decrease	−1.43	Deleterious	−5.36
rs769331894	F177 L	Deleterious	0	Probably damaging	1	Large decrease	−1.02	Deleterious	−5.27
rs769805145	P288A	Deleterious	0	Probably damaging	1	Large decrease	−1.34	Deleterious	−7.62
rs770087890	G197A	Deleterious	0	Probably damaging	0.999	Large decrease	−1.07	Deleterious	−5.27
rs771733089	R83C	Deleterious	0	Probably damaging	0.997	Large decrease	−0.89	Deleterious	−2.72
rs772416503	P496R	Deleterious	0	Probably damaging	1	Large decrease	−0.7	Deleterious	−7.58
rs772448566	F352 L	Deleterious	0	Probably damaging	1	Large decrease	−1.17	Deleterious	−5.57
rs772855552	A351T	Deleterious	0	Probably damaging	0.999	Large decrease	−0.75	Deleterious	−3.44
rs773471238	V379G	Deleterious	0	Probably damaging	1	Large decrease	−2.32	Deleterious	−5.49
rs773908263	P81S	Deleterious	0	Probably damaging	1	Large decrease	−1.86	Deleterious	−6.31
rs774139207	E299D	Deleterious	0	Probably damaging	1	Large decrease	−0.77	Deleterious	−2.95
rs775542391	L114R	Deleterious	0	Probably damaging	1	Large decrease	−1.76	Deleterious	−4.89
rs775816150	T374P	Deleterious	0	Probably damaging	1	Large decrease	−0.81	Deleterious	−5.09
rs776561903	P55A	Deleterious	0	Probably damaging	0.997	Large decrease	−1.41	Deleterious	−4.57
rs780165694	Y76C	Deleterious	0	Probably damaging	1	Large decrease	−1.09	Deleterious	−4.34
rs781136336	L526P	Deleterious	0	Probably damaging	0.999	Large decrease	−1.11	Deleterious	−3.93
rs781149866	R368H	Deleterious	0	Probably damaging	1	Large decrease	−1.17	Deleterious	−3.47
rs794726877	G51D	Deleterious	0	Probably damaging	1	Large decrease	−0.59	Deleterious	−5.45
rs794727017	P510R	Deleterious	0	Probably damaging	1	Large decrease	−0.62	Deleterious	−5.79
rs794727896	T388 K	Deleterious	0	Probably damaging	1	Large decrease	−0.74	Deleterious	−4.24
rs866224971	R447C	Deleterious	0	Probably damaging	0.999	Large decrease	−1.12	Deleterious	−3.3
rs869025584	L218P	Deleterious	0	Probably damaging	0.996	Large decrease	−1.63	Deleterious	−5.03
rs875989946	W175R	Deleterious	0	Probably damaging	1	Large decrease	−1.23	Deleterious	−13.14
rs375819348	P493R	Deleterious	0	Probably damaging	1	Large decrease	−0.82	Deleterious	−8.33
rs767140903	P302R	Deleterious	0	Probably damaging	1	Large decrease	−0.95	Deleterious	−7.79

All 93 SNPs identified were further analyzed by PANTHER, SNPs&GO and PHD-SNP. PANTHER characterizes the effect of amino acid variation on protein function via HMM based statistical modeling. PANTHER can classify proteins by function, adding another layer of complexity to refine SNP prediction. SNPs&GO predicts the log-odd (LGO) score from the GO data base by placing the similar proteins in the same dataset. PHD-SNP is an SVM-based classifier, trained over a million amino acid polymorphism datasets using supervised training. Out of the 93 SNPs, 28 were predicted to be disease-associated by three methods (Table 2).

**Table 2:** List of 28 nsSNP predicted as associated with disease by PHD-SNP, PANTHER and SNP&GO

SNP ID	AA change	PHD-SNP	Probability	PANTHER	Probability	SNP&GO	Probability
rs76722191	V322E	Disease	0.718	Disease	0.712	Disease	0.716
rs121965021	P533R	Disease	0.635	Disease	0.827	Disease	0.671
rs121965029	R89Q	Disease	0.841	Disease	0.609	Disease	0.745
rs121965033	L346R	Disease	0.76	Disease	0.792	Disease	0.663
rs148789453	L238Q	Disease	0.777	Disease	0.553	Disease	0.513
rs200448421	R628P	Disease	0.817	Disease	0.529	Disease	0.634
rs368454909	D349N	Disease	0.723	Disease	0.603	Disease	0.54
rs369090960	G265R	Disease	0.12	Disease	0.841	Disease	0.63
rs371397270	D349G	Disease	0.754	Disease	0.663	Disease	0.593
rs373037758	L256P	Disease	0.881	Disease	0.795	Disease	0.801
rs374779600	P533S	Disease	0.539	Disease	0.718	Disease	0.579
rs587779401	Y625C	Disease	0.722	Disease	0.867	Disease	0.724
rs748239393	F287C	Disease	0.668	Disease	0.862	Disease	0.628
rs750496798	R363C	Disease	0.764	Disease	0.903	Disease	0.701
rs753308650	G168R	Disease	0.91	Disease	0.841	Disease	0.846
rs754154200	E182K	Disease	0.772	Disease	0.591	Disease	0.702
rs754876066	T194P	Disease	0.747	Disease	0.732	Disease	0.628
rs757706461	P183S	Disease	0.619	Disease	0.718	Disease	0.602
rs762623046	R166T	Disease	0.739	Disease	0.547	Disease	0.639
rs772416503	P496R	Disease	0.606	Disease	0.827	Disease	0.568
rs773908263	P81S	Disease	0.725	Disease	0.688	Disease	0.667
rs775542391	L114R	Disease	0.818	Disease	0.786	Disease	0.743
rs775816150	T374P	Disease	0.771	Disease	0.732	Disease	0.538
rs780165694	Y76C	Disease	0.646	Disease	0.703	Disease	0.522
rs794726877	G51D	Disease	0.74	Disease	0.79	Disease	0.704
rs875989946	W175R	Disease	0.866	Disease	0.839	Disease	0.831
rs767140903	P302R	Disease	0.673	Disease	0.827	Disease	0.678
rs375819348	P493R	Disease	0.681	Disease	0.827	Disease	0.633

### Biophysical validation and 3D structure analysis of missense SNPs

Based on the in silico analyses performed, 28 SNPs were selected for biophysical analysis using NetSurfP. The location and the type of a mutated residue can affect the stability of the protein by decreasing the solvent accessibility of a residue decreases. NetSurfP Z-score allows for the identification of the most reliable predictions for both buried and exposed amino acids. Out of 28 SNPs, a huge drift in the Z-score was observed for 5 SNPs (Table 3).

**Table 3:** Surface accessibility of native and mutant IDUA variants that are selected for structural analysis

SNP ID	AA	AA position	RSA	ASA	Z-fit score for RSA prediction	Class assignment
rs121965021	P	533	0.341	48.43	−1.149	Buried
	R		0.344	78.73	−0.651	Buried
rs371397270	D	349	0.241	34.699	−0.488	Buried
	G		0.392	30.819	−1.405	Exposed
rs775816150	T	374	0.095	13.149	−1.852	Buried
	P		0.233	33.134	−1.134	Buried
rs772416503	P	496	0.105	14.928	0.247	Buried
	R		0.164	37.533	−0.253	Buried
rs121965033	L	346	0.031	5.603	0.342	Buried
	R		0.108	24.09	−0.954	Buried

*AA* amino acid, *RSA* relative surface accessibility, *ASA* absolute surface accessibility

**Fig. 1:**
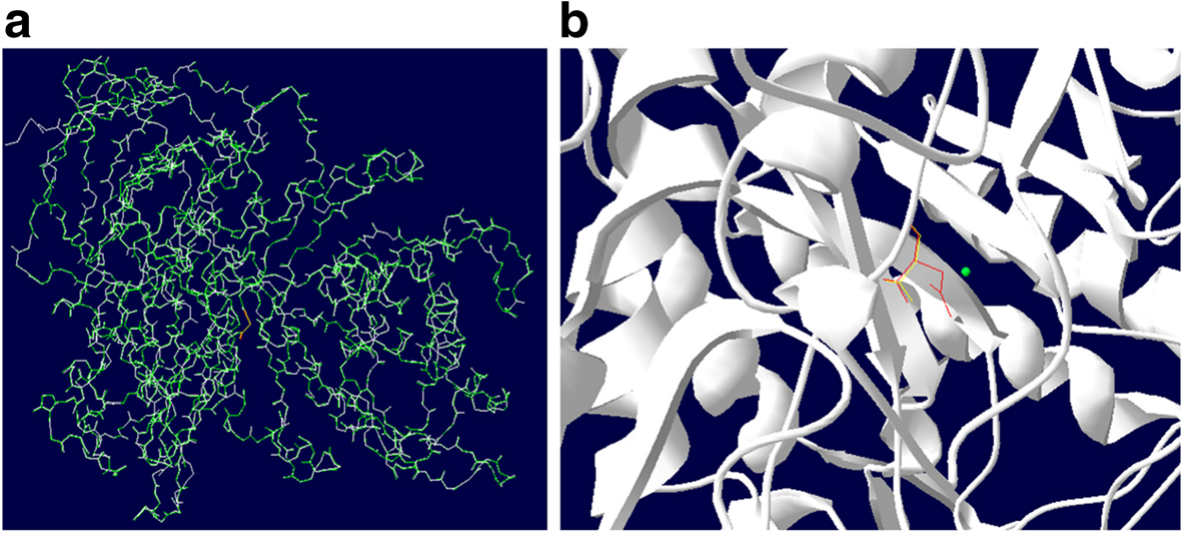
Superimposed structure of native protein with modeled mutant protein for D349G. **a** Overall structure of the superimposed model. Native protein in white (cartoon shape), mutant protein in green, wild type residue (Asp349) in red, and mutated residue (Gly349) in yellow. **b** close-up view of the superimpose model. Main protein backbone in white, wild type reside (Asp349) in red, mutated residue (Gly349) in yellow, a chloride anion in green

**Fig. 2:**
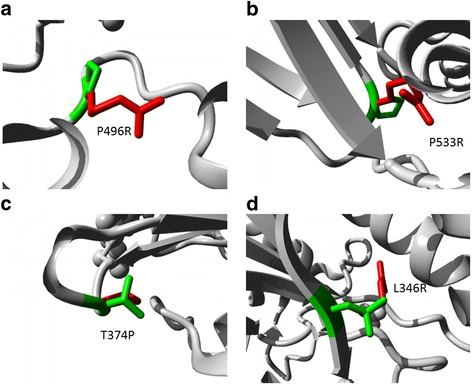
Close-up view of superimposed structure of native and mutant residues (**a** P496R; **b** P533R; **c** T374P; **d** L346R). The main protein core is shown in white color while the wild type and mutated residues are shown in red and yellow color, respectively

To analyze the 3D structural change introduced by these 5 SNPs, we performed structural analysis by comparing the native and mutant protein structures. Briefly, the native structure of IDUA was extracted from Protein Data Bank (ID 3 W81). Single amino acid substitution and superimposition of native and mutated structures were examined using Swiss-PDB viewer, and their degree of similarity was measured as the RMSD value. RMSD values between native and each mutant structure are <0.5 Å, indicating a minor structural change caused by the SNP. An illustration of overall superimposition by Swiss-PDB viewer is shown in Fig. 1, while detailed structural changes in Fig. 2. Total energy values of native structure and 5 mutant structures were calculated after energy minimization by NOMAD_Ref and summarized in Table 4. The total energy of three mutant models (L346R, P496R and P533R) is significantly higher than that of the native model, indicating that the mutation decreases the protein stability.

**Table 4:** Total energy of native and mutant structures after energy minimization

SNP ID	AA change	Total energy after minimization (KJ/mol)
	Native	−58,850
rs121965033	L346R	−57,705
rs772416503	P496R	−54,038
rs121965021	P533R	−22,157
rs775816150	T374P	−58,766
rs371397270	D349G	−58,730

Specifically, rs772416503 leads to conversion of proline into arginine at position 496 (P496R). The hydrophobic environment around Pro496 leaves no room for a bulky polar residue (arginine). This mutation (P496R) may interfere with the placement of Asn372 glycan over the active site, and thereby affect enzyme catalytic activity. Rs371397270 leads to conversion of aspartic acid into glycine at position 349 (D349G). Asp349 is located in triosephosphateisomerase (TIM) barrel active site and interacts with substrate. Besides, since glycine is smaller than aspartic acid, the mutation will cause an empty space in the core of the protein. The charge of the buried wild-type residue is also lost due to this mutation. Therefore, D349G will also cause loss of hydrogen bonds in the core of the protein and thereby disturb correct folding. Rs121965021 (P533R) is located in the β sandwich. Prolines are known to have a very rigid structure, sometimes forcing the backbone in a specific conformation. P533R may disturb this special conformation and destabilize the β sandwich domain by introducing the side chain of arginine. Besides, only the wild type residue proline is found at this position. Mutation of a 100% conserved residue is usually damaging for the protein. Rs121965033 (L346R), located in the TIM barrel, may cause steric hindrance and destabilize active site confirmation. The mutant residue (Arg) introduces a charge in a buried residue (Leu) which affects protein folding. Besides, since Leu346 is buried in the core of the protein, Arg is bigger and probably will not fit. This mutation will cause loss of hydrophobic interactions in the core of the protein. Rs775816150 (T374P) is located at Thr374, a conserved N glycosylation site. It has also been shown that N-glycans are essential for substrate binding and catalytic activity of IDUA [28]. Therefore, this mutation (T374P) may lead to decrease or loss of catalytic activity of IDUA.

### Establishment and evaluation of SNPs prediction algorithm

By integrating outcomes of the bioinformatics tools listed in Section 3.1, a prediction algorithm (SAAMP: Single Amino Acid Mutation Predictor) with a pathogenic index (PI) was developed. PI is defined as percentage of ‘damaging’ predictions from these 7 bioinformatics tools. The higher the PI is, the more pathogenic the SNP is. The cut-off value is set at 0.43. When PI is ≥0.43 (larger than or equal to 3 damaging related predictions), the mutation is defined as ‘pathogenic’, otherwise it is ‘benign’. A total of 81 known disease-associated missense mutations and 15 known benign polymorphisms of IDUA were analyzed by these bioinformatics tools, and the PI of each mutation was calculated. By assessing false positives and false negatives, a sensitivity of 94% and a specificity of 80% were reached. The false positives and false negatives were evaluated manually, however, no significant patterns were observed. It might be due to the differences in methodologies utilized by these in silico tools. Alternatively, when the cut-off value is set as 0.57 (larger than or equal to 4 damaging related predictions), a sensitivity of 79% and a specificity of 93% was calculated. In order to increase the probability of identifying pathogenic mutations and minimize the risk of neglecting patients, high sensitivity is preferable and the cut-off value of 0.43 is recommended.

### Functional SNPs in UTRs identified by UTSscan and PolymiRTs

All of the 177 UTR SNPs were analyzed using UTRscan. It has been shown that polymorphisms in 3′ UTR region can affect the gene expression pattern during mRNA translation, while the polymorphisms in 5′ UTR region affect the RNA half-life by altering the polyadenylation [28, 29]. After comparing the functional elements for each UTR SNP, we predicted that 6 SNPs in 5′ UTR are related to the functional pattern changes including internal ribosome entry site (IRES) and 15-Lipoxygenase Differentiation Control Element (15-LOX-DICE) (Table 5). The IRES is involved in internal mRNA ribosome binding, which allows for translation when the conventional mechanism of translation is ineffective. 15-LOX-DICE is a multifunctional cis-element found in the 3′ UTR of numerous eukaryotic mRNAs. 15-LOX-DICE binds heterogeneous nuclear ribonucleoproteins (hnRNP) E and K, thus mediating mRNA stabilization and translational control. Among 19 SNPs in 3′ UTR region of IDUA gene, only one SNP (rs733349) is predicted to disrupt 13 non-conserved miRNA sites (ancestral allele with support <2) and create 8 new miRNA sites (Table 6).

**Table 5:** List of mRNA UTR SNPs that were predicted to be of functional significance by UTRscan server

SNP ID	Nucleotide change	UTR position	Functional element change
rs577729544	G/A	5’	IRES → no pattern
rs200237798	G/A	5’	IRES → no pattern
rs372934646	C/A	5’	IRES → no pattern
rs530362790	G/A	5’	No pattern → 15-LOX-DICE
rs765255638	G/T	5’	IRES → no pattern
rs775542391	T/G	5’	IRES → no pattern

*15-LOX-DICE* 15-Lipoxygenase Differentiation Control Element, *IRES* internal ribosome entry site

**Table 6:** Prediction result of PolymiRTS database

SNP ID	miR ID	Conservation	miRSite	Function class
rs733349	hsa-miR-128-3p	1	ggctgCACTGTGc	N
	hsa-miR-148a-3p	1	ggcTGCACTGtgc	N
	hsa-miR-148b-3p	1	ggcTGCACTGtgc	N
	hsa-miR-152-3p	1	ggcTGCACTGtgc	N
	hsa-miR-216a-3p	1	ggctgCACTGTGc	N
	hsa-miR-3681-3p	1	ggctgCACTGTGc	N
	hsa-miR-3944-5p	1	gGCTGCACtgtgc	N
	hsa-miR-7156-3p	1	GGCTGCActgtgc	N
	hsa-miR-4436b-3p	1	ggCTGCCCTgtgc	C
	hsa-miR-4632–5p	1	ggCTGCCCTgtgc	C
	hsa-miR-6735-5p	1	ggCTGCCCTgtgc	C
	hsa-miR-6879-5p	1	ggCTGCCCTgtgc	C
	hsa-miR-7843-5p	1	ggCTGCCCTgtgc	C

Conservation: Occurrence of the miRNA site in other vertebrate genomes in addition to the query genome. By clicking the hyperlink, the users can examine the genomes in which this miRNA target site occurs. miRSite: Bases complementary to the seed region are in capital letters. Explanation of the function class is listed as followed. D: The derived allele disrupts a conserved miRNA site (ancestral allele with support > = 2). N: The derived allele disrupts a nonconserved miRNA site (ancestral allele with support <2). C: The derived allele creates a new miRNA site. O: The ancestral allele cannot be determined

### Phenotypic severity prediction of known disease-associated mutations

Proper and timely treatment allocation based on phenotype severity prediction is essential for benefits of patients. The aforementioned bioinformatics tools are not designed specifically for MPS I disease, and are unable to predict the phenotype severity (Hurler, Hurler-Scheie or Scheie). Therefore, an extensive review of previous publications reporting pathogenic mutations of IDUA was conducted to make inferences about phenotype severity. A total of 185 mutations have been identified, including 86 missense mutations, 22 nonsense mutations, 45 deletions/insertions and 32 splicing mutations. By analyzing the phenotypes and mutations on both alleles of patients from the original reports, phenotype prediction of each mutation was conducted manually. Four general assumptions were used as followed: 1) only when both alleles are predicted to be severe, the phenotype is Hurler; 2) if one allele is predicted to be mild (intermediate) while the other severe, the phenotype is Scheie (Hurler-Scheie); 3) if both alleles are intermediate, the phenotype is Hurler-Scheie or Scheie; 4) even only one allele is predicted to be mild, the phenotype is Scheie (illustrated in Additional file 1: Fig. S1). Further, the crystal structure of IDUA has been elucidated [30, 31], which was used to further confirm and rectify the predictions made in Tables 7 and 8. Notably, due to lack of enough information and consensus of phenotype severity, it is difficult to make a comprehensive evaluation of reliability of the original reports. Therefore, we highlighted the severity predictions with relatively low reliability with ‘*’ in Tables 7 and 8. All identified nonsense mutations are severe. W402X and Q70X are the most common nonsense mutations found in patients from different ethnic groups. Out of 32 splicing mutations, 20 are predicted to be severe, 5 intermediate, 1 mild (IVS5-7G > A) and 4 with unknown effects. Out of 45 deletions/insertions, 38 are predicted to be severe, which is reasonable due to the usual consequence of frame shift. However, there might be some exceptions: 396insAC, c.1593delG, and 1995del11 with Hurler-Scheie or Scheie phenotype. 1995del11 is in the final exon of IDUA, which may lead to residual enzyme activity. c.1593delG was found to be in trans with a missense mutation (deduced to be severe from multiple reports) in a Hurler-Scheie patient [32]. However, although this patient is defined as Hurler-Scheie, delayed mental development was observed. Therefore, this patient may actually have Hurler disease, which will make 1592delG ‘severe’. Similarly, additional evidence is required to determine the phenotypic severity of 396insAC. Missense mutations are the least severe type, with only 31 out of 86 are predicted to be severe. P533R is the most frequent but complicated missense mutation, which has been found in the homozygous state in patients with Hurler, Hurler-Scheie and Scheie phenotypes. Due to convenience consideration, the nomenclature of mutations in this study still uses the old names as reported in previous publications. However, as suggested in the current guideline on nomenclature [33], it will be important to follow this guideline to name newly identified mutations.

**Table 7:** Phenotype/genotype correlation of missense and nonsense mutations in IDUA gene

Mutation	Phenotype prediction	Mutation	Phenotype prediction	Mutation	Phenotype prediction
Y76C	mild	M504T	intermediate	V620F	severe
R89W	mild	L535F	intermediate	R628P	severe
R89Q	mild, intermediate	R619G	intermediate	X654C	severe
A160D	mild, intermediate	W626R	intermediate	L421P	unknown
C205Y	mild	X654G	intermediate	L578Q	unknown
G219E	mild, intermediate	X654R	intermediate, severe	G168 V	unknown
H240R	mild	M1 T	severe	F52 L	unknown
E276K	mild, intermediate	G51D	severe	L396P	unknown
W306 L	mild, intermediate	A75T	severe	P533R	unknown
A319V	mild, intermediate	T103P	severe	H33P	unknown
L346R	mild, intermediate	M133I	severe	A79V	unknown
N348 K	mild^a^	T141S	severe	G197S	unknown
N350I	mild, intermediate	F177S	severe	W41X	severe
Q380R	mild, intermediate	E182D	severe	C53X	severe
R383H	mild, intermediate	E182K	severe	Q60X	severe
T388R	mild	P183R	severe	Q63X	severe
S423R	mild, intermediate	D203N	severe	Y64X	severe
R492P	mild	G208D	severe	Q70X	severe
S633 L	mild, intermediate	G208 V	severe	Y167X	severe
M1I	intermediate	L218P	severe	Y201X	severe
A75P	intermediate	L237R	severe	E274X	severe
H82P	intermediate	L238R	severe	E299X	severe
G84R	intermediate^a^	I270S	severe	Q310X	severe
E178K	intermediate	L308P	severe	Y343X	severe
T179R	intermediate, severe	D315Y	severe	W402X	severe
F188 L	intermediate^a^	A327P	severe	E404X	severe
G197D	intermediate	D349N	severe	W420X	severe
L238Q	intermediate	D349Y	severe	Q561X	severe
S260F	intermediate^a^	R363C	severe	Y581X	severe
G265R	intermediate	T366P	severe	Q584X	severe
R363H	intermediate	T374 N	severe	R619X	severe
T364 M	intermediate	P385R	severe	R621X	severe
A436P	intermediate	R489P	severe	W626X	severe
G409R	severe	P496R	severe	R628X	severe
L490P	intermediate	P533L	severe		
P496L	intermediate	F602I	severe		

^a^was added to predictions with relatively low reliability

**Table 8:** Phenotype/genotype correlation of splicing, deletions and insertions mutations in IDUA gene

Mutation	Phenotype prediction	Mutation	Phenotype prediction	Mutation	Phenotype prediction
134del12	severe	c.1147dupG	severe	IVS4-1G > A	intermediate, severe
153delC	severe	c.1166_1171dup	severe	IVS4 + 1G > A	intermediate
229del3	severe	c.1190-1delG	severe	IVS5-7G > A	mild
252insC	severe	c.1225dupG	severe	IVS5 + 1G > A	severe
c.349delT	unknown	c.1244-1271del27	severe	IVS6 + 1G > C	severe
396insAC	mild^a^	1251delC	severe	IVS6 + 1G > T	severe
468del3	severe	1277ins9	severe	IVS7-4G > A	severe
486del6	unknown	1352delG	severe	IVS7 + 2 T > C	unknown
c.574delT	severe	c.1398delC	severe	IVS8-1G > A	severe
628del5	severe	c.1589insGC	severe	IVS8 + 4G > A	intermediate
c.657dupG	severe	c.1593delG	intermediate^a^	IVS8 + 5G > A	intermediate, severe
668insGCG	severe	1702delG	severe	IVS9 + 1G > T	severe
682insAC	severe	1783del11	severe	IVS9 + 2 T > G	unknown
702ins10del22	severe	c.1805delTinsGAACA	severe	IVS11-G > T	severe
704ins5	severe	1839del29	severe	IVS11 + 5G > A	severe
740delC	severe	1902del2	severe	IVS11 + 5G > C	severe
747delG	severe	c.1918_1927del10	intermediate^a^	3308del12	intermediate
755del5	severe	1995del11	intermediate^a^	IVS12 + 1G > A	severe
c.826_828del3	severe	D444/445	mild	IVS12 + 2 T > G	severe
c.854delC	severe	c.1-2C > G	severe	IVS12 + 2 T > A	unknown
c.883dupC	severe	IVS2-1G > C	severe	IVS12 + 3G > C	severe
c.956_972 + 9delinsTA	severe	IVS2-3C > G	unknown	IVS12 + 4C > T	intermediate
964delC	severe	IVS2 + 1G > A	intermediate	IVS12 + 5G > A	severe
974ins12	mild	IVS2 + 6C > T	severe	IVS12 + 5G > C	unknown
c.1045_1047del3	severe	IVS3-2A > G	severe	IVS12 + 6 T > A	severe
1132del6	severe	IVS3 + 1G > A	severe		

^a^was added to predictions with relatively low reliability

## Discussion

The identification of SNPs responsible for specific phenotypes with molecular approaches can be expensive and time-consuming [34]. Therefore, computational approaches can be of great help by narrowing down the number of missense mutations to be screened in genetic association studies and advancing the understanding of functional and structural aspects of the protein. Since existing in silico methods have widely varying performance, no single method could be considered as the best and most accurate for predicting functional SNPs. Therefore, a combination of methods based on evolutionary information, protein structure and functional parameters were used in order to increase the prediction accuracy. Notably, there is no specific order for using these bioinformatics tools.

In this study, significant concordance was observed between the functional consequences of nsSNPs predicted by various combinations of the tools. Out of 201 missense nsSNPs predicted to be ‘deleterious’ by SIFT, 149 (74%) were also predicted to be ‘probably damaging’ by PolyPhen. Out of 285 missense nsSNPs, 93 (47%) were predicted to be ‘damaging’ by SIFT, PolyPhen, I-Mutant and PROVEAN. Then, these 93 nsSNPs were analyzed by PHD-SNP, SNPs&GO and PANTHER, and 28 (30%) were predicted to be disease-associated. Further, the SNPs predicted by these in silico approaches were well supported by experimental and clinical reports. We cross-referenced the results of in silico analysis and previously identified disease-associated mutations in HGMD. Out of 28 missense SNPs (Table 2) predicted, 18 (64%) have been identified to be disease-associated in the HGMD. These results demonstrated that implementations of different algorithms could serve as reliable and powerful tools for prioritizing candidate functional nsSNPs.

**Fig. 3:**
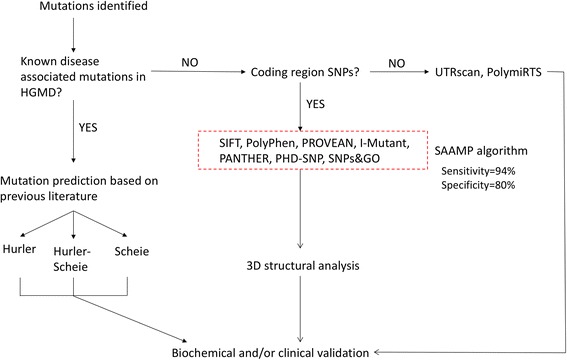
Step-by-step guideline for phenotype prediction by in silico analysis

Based on the results in this study, a step-by-step guiding model for phenotype prediction of MPS I disease was established (Fig. 3). When a mutation is identified, 1) if it is a known disease-associated mutation, refer to Tables 7 and 8 for phenotype severity prediction; 2) if not, conduct the in silico analysis of coding region SNPs and UTR SNPs, respectively. As discussed previously [35], even multiple lines of computational evidence only count as a single supporting criterion for classifying variants as pathogenic or benign. Therefore, further confirmation should be conducted through biochemical and/or clinical analyses. This model will be of great use by providing a valid, time-saving, cheap and easy-to-use method for phenotype prediction for a variety of diseases including MPS I. Admittedly, there are some limitations of this model. First, the in silico analysis is not sensitive enough for phenotype severity prediction because there are no algorithms specifically designed for this purpose. Second, the 3D structural analysis relies on the availability of 3D structure, rendering it difficult for analyzing proteins without solved structures. In this case, homology modeling can be applied to bridge this gap by predicting unknown protein structures.

## Conclusions

In conclusion, structural and functional impacts of nsSNPs in the IDUA gene were predicted using powerful computational tools. By predicting the possible deleterious SNPs of IDUA gene, the number of SNPs screened in association with diseases can be narrowed down to those that are most likely to alter gene function. Further, a model of phenotype prediction for MPS I disease by a combination of bioinformatics tools is established, which will benefit diagnosis and treatment allocation of MPS I patients. In the future, it will be essential to optimize the SAAMP algorithm by integrating the scores from each method with more sophisticated statistical methods, and validate it in a broad array of genes.

## Additional file

Additional file 1: Figure S1. General assumptions for phenotype severity prediction. (PPTX 245 kb)

## Abbreviations

15-LOX-DICE: 15-Lipoxygenase Differentiation Control Element
GAG: glycosaminoglycans
HGMD: human gene mutation database
HMM: hidden Markov model
hnRNP: heterogeneous nuclear ribonucleoproteins
HOPE: Have yOur Protein Explained
IDUA: α-L-iduronidase
IRES: internal ribosome entry site
MPS I: mucopolysaccharidosis type I
nsSNPs: non-synonymous SNPs
PHD-SNP: Predictor of Human Deleterious Single Nucleotide Polymorphisms
PI: pathogenic index
PolyPhen: Polymorphism Phenotyping
PROVEAN: Protein Variation Effect Analyzer
RMSD: root mean square deviation
SAAMP: Single Amino Acid Mutation Predictor
SIFT: Sorting Intolerant From Tolerant
SNPs: single nucleotide polymorphisms
SNPs&GO: Single Nucleotide Polymorphism Database & Gene Ontology
subPSEC: substitution position-specific evolutionary conservation score
SVM: support vector machine
TIM: triosephosphateisomerase
