# Supplementary figures and images for: Phenotype prediction for mucopolysaccharidosis type I by in silico analysis
Source: Orphanet J Rare Dis. 2017 Jul 4;12:125. doi: 10.1186/s13023-017-0678-1 (PMC5496269; doi:10.1186/s13023-017-0678-1)

## Slide 1
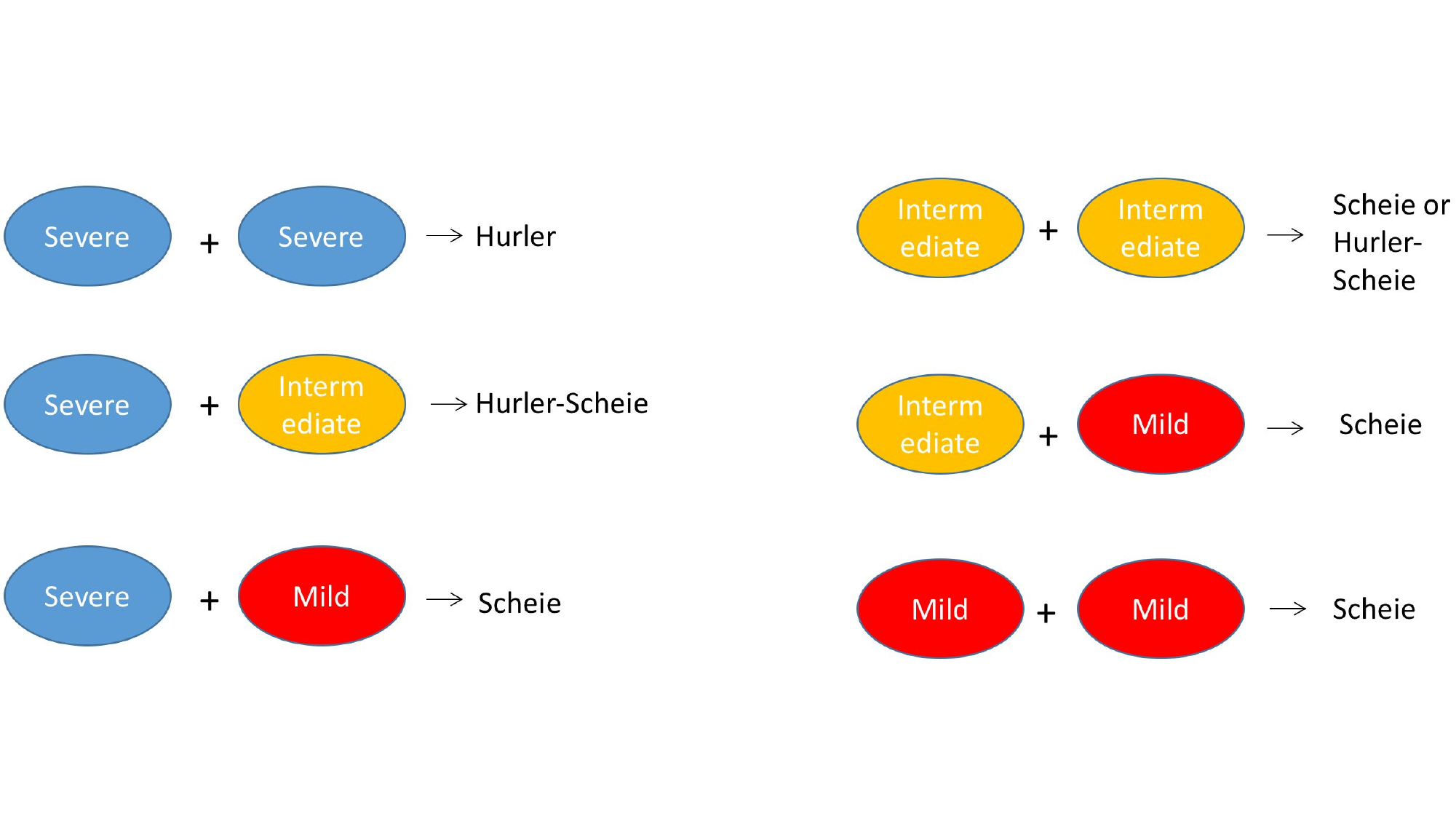

Supplement: Additional file 1: Figure S1. — General assumptions for phenotype severity prediction. (PPTX 245 kb) [file 13023_2017_678_MOESM1_ESM.pptx]
